# Rapid succession drives spring community dynamics of small protists at Helgoland Roads, North Sea

**DOI:** 10.1093/plankt/fbaa017

**Published:** 2020-05-14

**Authors:** Laura Käse, Alexandra C Kraberg, Katja Metfies, Stefan Neuhaus, Pim A A Sprong, Bernhard M Fuchs, Maarten Boersma, Karen H Wiltshire

**Affiliations:** 1 ALFRED-WEGENER-INSTITUT, HELMHOLTZ-ZENTRUM FüR POLAR- UND MEERESFORSCHUNG, BIOLOGISCHE ANSTALT HELGOLAND, 27498 HELGOLAND, Germany; 2 ALFRED-WEGENER-INSTITUT, HELMHOLTZ-ZENTRUM FüR POLAR- UND MEERESFORSCHUNG, 27570 BREMERHAVEN, Germany; 3 HELMHOLTZ-INSTITUT FüR FUNKTIONELLE MARINE BIODIVERSITäT, 26129 OLDENBURG, Germany; 4 DEPARTMENT OF MOLECULAR ECOLOGY, MAX PLANCK INSTITUTE FOR MARINE MICROBIOLOGY, 28359 BREMEN, Germany; 5 UNIVERSITY OF BREMEN, 28359 BREMEN, Germany; 6 ALFRED-WEGENER-INSTITUT, HELMHOLTZ-ZENTRUM FüR POLAR- UND MEERESFORSCHUNG, WADDEN SEA STATION, 25992 LIST AUF SYLT, Germany

**Keywords:** phytoplankton, diversity, German bight, Illumina MiSeq sequencing, Long-Term Ecological Research (LTER)

## Abstract

The dynamics of diatoms and dinoflagellates have been monitored for many decades at the Helgoland Roads Long-Term Ecological Research site and are relatively well understood. In contrast, small-sized eukaryotic microbes and their community changes are still much more elusive, mainly due to their small size and uniform morphology, which makes them difficult to identify microscopically. By using next-generation sequencing, we wanted to shed light on the Helgoland planktonic community dynamics, including nano- and picoplankton, during a spring bloom. We took samples from March to May 2016 and sequenced the V4 region of the 18S rDNA. Our results showed that mixotrophic and heterotrophic taxa were more abundant than autotrophic diatoms. Dinoflagellates dominated the sequence assemblage, and several small-sized eukaryotic microbes like Haptophyta, Choanoflagellata, Marine Stramenopiles and Syndiniales were identified. A diverse background community including taxa from all size classes was present during the whole sampling period. Five phases with several communities were distinguished. The fastest changes in community composition took place in phase 3, while the communities from phases 1 to 5 were more similar to each other despite contrasting environmental conditions. Synergy effects of next-generation sequencing and traditional methods may be exploited in future long-term observations.

## INTRODUCTION

Planktonic eukaryotic microbes as encompassed by the term “phytoplankton” represent a diverse array of plankton groups of all size classes including pico- and nanoplankton. They comprise the most frequent autotrophic groups such as diatoms, coccolithophores, green algae and cyanobacteria, but also dinoflagellates, which contain autotrophs, as well as heterotrophs and mixotrophs (Sournia *et al*., 1991; Simon *et al*., 2009). Photoautotrophic phytoplankton is responsible for half of the global primary production (Field *et al*., 1998). Primary producers are important not only as a food source for their consumers but also for bacterial plankton, as bacteria can feed on their excretory products or internal storage compounds after cell death in the form of dissolved or particulate organic matter (Sherr and Sherr, 2002). Microbial mixotrophic and heterotrophic consumers (e.g. choanoflagellates, cryptophytes, dinoflagellates) can feed on the heterotrophic bacterioplankton (bacterivorous protists) or on phytoplankton (herbivorous protists) and are themselves food for the higher trophic zooplankton. Thus, planktonic eukaryotic microbes play an important role in the so-called microbial loop (Azam *et al*., 1983; Sherr and Sherr, 2002; Caron and Hu, 2019). All size classes, including nano- and picoplankton, are present at different trophic levels of the planktonic community. However, thus far, these are barely identifiable to species level by traditional microscopic methods because of their miniscule size and uniform morphology.

On a global scale, phytoplankton growth periods vary depending on the climate zone; while long growth periods with low biomass occur mostly in tropical and subtropical regions, short growing periods with high biomass have been recorded for high-latitude regions (Racault *et al*., 2012). How different components of the eukaryotic microbial community are present throughout the year in the North Sea and at Helgoland is governed by many abiotic and biotic factors (Reid *et al*., 1990; Wiltshire *et al*., 2015), and species often show distinct seasonal succession patterns (Scharfe and Wiltshire, 2019). During winter there is not much light available, and temperature is low in temperate regions; however, towards the end of winter, nutrient concentrations are high due to remineralization. This leaves optimal conditions specifically for autotrophic organisms like diatoms to bloom once temperature and, more importantly, light availability increase. These spring blooms neither manifest with the same species, nor are they dominated by one species throughout the bloom. Instead they are often a mixture of species at the beginning and show a distinct succession on different timescales (Lewandowska et al., 2015; Scharfe and Wiltshire, 2019). Traditionally in spring blooms in temperate regions, e.g. at the Long-Term Ecological Research (LTER) site Helgoland Roads in the German Bight (Wiltshire *et al*., 2010), diatoms are considered as the major phytoplankton bloom components, showing distinct and massive blooming patterns (Mieruch *et al*., 2010). The bloom of autotrophic phytoplankton is then typically followed by dinoflagellates, heterotrophic plankton or larger zooplankton such as copepods (Lewandowska *et al*., 2015; Wiltshire *et al*., 2015).

The knowledge of spring bloom dynamics in specific regions can be validated and extended by implemented time series like the Continuous Plankton Recorder Survey (Reid *et al*., 2003; McQuatters-Gollop *et al*., 2015), the L4 coastal time-series station (Harris, 2010) or Helgoland Roads LTER (Wiltshire and Manly, 2004; Wiltshire *et al*., 2015; Scharfe and Wiltshire, 2019). One potential problem, however, is that traditional time series currently rely on microscopy techniques, such as the Utermöhl method, which is time-consuming and limited by the size of organisms (Stern *et al*., 2018). This means that the smallest organisms cannot be assigned to taxonomic level accurately (Culverhouse, 2015). Therefore, especially small protists are barely investigated due to the resolution limit of the identification methods used in traditional long-term observations.

New molecular methods, and especially next-generation sequencing (NGS), could have a high potential for very detailed monitoring (Ebenezer *et al*., 2012; Stern *et al*., 2018) as these molecular methods reliably capture the entire phytoplankton community including nano- and picoplanktonic components. For example, seasonality patterns could be found for at the Adventfjorden time-series station using 454 sequencing (Marquardt *et al*., 2016) and in the Mediterranean Sea using Illumina sequencing (Giner *et al*., 2019). Seasonal patterns as well as diel shifts in activity could be found using the V4 region of RNA and DNA in Illumina sequencing in the North Pacific (Hu *et al*., 2016, 2018). Other studies have been conducted, focusing on different European coastal waters like the L4 time-series station in the Western English Channel (Taylor and Cunliffe, 2014), several stations along the European coast within the BioMarKs project (Logares *et al*., 2014; Massana *et al*., 2014, 2015) or estuaries, e.g. in the eastern English Channel (Bazin *et al*., 2014). Some studies only focus on certain taxa, e.g. uncultured marine heterotrophic flagellates (Logares *et al*., 2012) or Chlorophyta (Tragin *et al*., 2018). With regard to prokaryotic monitoring, several NGS studies were conducted at Helgoland (Lucas *et al*., 2015, 2016; Teeling *et al*., 2016; Chafee *et al*., 2018). While several studies have been conducted in the general North Atlantic at large, only a few studies focusing on specific groups of small-sized eukaryotic protists have been done using other molecular methods, which are focused specifically on Helgoland (Medlin *et al*., 2006, 2017; Gescher *et al*., 2008; Knefelkamp, 2009; Metfies *et al*., 2010).

This study aims to (1) understand the community structure and dynamics of eukaryotic protists including the pico- and nanoplankton fraction during spring and (2) discover if the typical spring bloom succession of diatoms and dinoflagellates can be detected using NGS data at Helgoland Roads from 15 March to 31 May 2016. (3) We also aim to relate abiotic dynamics in the water column to taxonomic group shifts in the community during the spring bloom based on a much more detailed assessment of phytoplankton biodiversity.

## MATERIALS AND METHODS

In total, we took 50 plankton samples during spring 2016, analysed these samples using next-generation sequencing (18S) and investigated successional patterns.

### Study site and sampling

Sampling was conducted at the Helgoland Roads LTER sampling site at the station “Kabeltonne” (54°11.03′ N, 7°54.00′E, Germany) (Wiltshire and Dürselen, 2004). The sampling site is situated between the main island and the dune island of Helgoland. The generally well-mixed water column fluctuates between 6 and 10 m depth, depending on the tides (Callies and Scharfe, 2015). Samples were taken from 1 m depth between 15 March and 31 May 2016. Sampling frequency was work-daily, according to the LTER sampling. About 1 L of seawater was sequentially filtered using 10 μm polycarbonate filters (PC), 3 μm PC filters and 0.2 μM polyvinylidene fluoride filters (Millipore, Schwalbach, Germany) to obtain the whole prokaryotic and eukaryotic plankton community (Teeling *et al*., 2016). Secchi depth and temperature were measured directly in the water at the sampling site. Other parameters, including salinity and nutrients such as silicate, phosphate and inorganic nitrogen using the methods of Grasshoff (1976), were measured in the laboratory according to the LTER protocols (Hickel *et al*., 1993; Wiltshire *et al*., 2008, 2010). Daily observations of sunshine duration in hours were downloaded from the Deutscher Wetterdienst, Climate Data Centre (2019). To check whether the spring of 2016 showed a typical phytoplankton community succession of diatoms followed by dinoflagellates as observed in the LTER, we used total diatom and total dinoflagellate counts and chlorophyll *a* measured by HPLC modified after Zapata et al. (2000) from 1st March to 31stMay.

### DNA extraction and pooling of samples

The DNA extraction from 0.2 μm filters was conducted as described previously at the Max Planck Institute for Marine Microbiology (Bremen, Germany) (Sapp *et al*., 2007). In short, lysozyme (1 mg mL^−1^) and sodium dodecyl sulphate (1%) were used for cell lysis; DNA was extracted with a phenol/chloroform/isoamyl alcohol mixture (25:24:1) and precipitated with isopropanol, before the DNA extracts were eluted in sterile water. This fraction, which was previously used for 16S analysis, was then added to the other fractions to include all potential eukaryotes in all size ranges. The DNA from the 10 and 3 μm filters was extracted following the manual of the Macherey–Nagel NucleoSpin® Plant II Kit, and all extracts were stored at −20°C. To include the whole eukaryotic plankton community from all size classes, equal volumes of the DNA extracts of the smallest size fraction (0.2 μm pore-size filters) were then pooled with the DNA extracts of the remaining size fractions (3 and 10 μm) to obtain one sample per sampling date. Measurement of nucleic acid content of the pools was conducted with a fluorometer (QuantiFluor® dsDNA System, Promega,USA).

### MiSeq™ Illumina sequencing

After pooling, the samples were prepared for MiSeq™ Illumina sequencing following the Nextera XT DNA Library Preparation protocol (Illumina, USA) with the following modifications: a fragment (V4 region) of the 18S ribosomal (r) DNA was amplified using KAPA HiFi HotStartReadyMix (Kapa Biosystems, Inc., USA) and the following primer set: 528iF (GCG GTA ATT CCA GCT CCA A) and 964iR (ACTTT CGT TCT TGA TYR R) (Fadeev *et al*., 2018). The success of this amplicon PCR was confirmed with gel electrophoresis using 2 μL of the PCR product. If no bands were detected, the amplicon PCR was repeated with an increased template volume (up to 5 μL). If this still was not sufficient to detect the respective band, five additional cycles were added to the original program (eight samples). Before library normalization and pooling, the DNA concentration was once again measured using a Quantus Fluorometer (Promega, USA) and diluted accordingly. Amplicon sequencing was then performed on an Illumina MiSeq™ sequencer (Illumina, USA), and about 6.3 million 2 × 300 bp paired-end reads were produced in total.

### Bioinformatics processing

Sequence processing, operational taxonomic unit (OTU) clustering and annotation were done with an internally developed pipeline at the Alfred Wegener Institute as described below (detailed description as Supplemental Material), wrapping common bioinformatics tools and “*GNU parallel*” (Tange, 2011) for fast and massive parallel workflow execution. The low-quality 3′-ends of the reads were trimmed by Trimmomatic, version 0.38 (Bolger *et al*., 2014), and the paired-ends were merged by PEAR, version 0.9.10 (Zhang *et al*., 2014). Cutadapt, version 1.17 (Martin, 2011), was used to adjust the sequence orientation and remove the forward and reverse primer matching sequence segments. Sequences were only kept if both primer matching segments could be detected. The remaining sequences were filtered by VSEARCH, version 2.3.0 (Rognes *et al*., 2016), and sequences were discarded, (i) if they were 50 bp longer or shorter than the median length of the targeted amplicon (376 bp), (ii) if they carried any ambiguity or (iii) if the expected base error (sum of all base error probabilities) of a sequence was above 0.5. Chimeric sequences were sample-wise predicted by VSEARCH, version 2.3.0, in de novo mode with default settings and removed from the sample files. Only samples with at least 10 000 sequences after filtering were considered for further analyses (49 out of 50 samples). The remaining 4.3 million sequences were clustered into OTUs by the tool swarm, version 2.1.8 (Mahé *et al*., 2014, 2015), with default settings. For each OTU the most abundant amplicon was selected as representative and taxonomically annotated with the default classifier implemented in mothur, version 1.38.1 (Schloss *et al*., 2009). As reference the Protist Ribosomal Reference database (PR2), version 4.10 (Guillou *et al*., 2013), was chosen and the confidence cut-off was set to a value of 90.

A conservative threshold of 0.005% (of total reads) after Bokulich *et al*. (2013) was applied to the remaining 37 608 OTUs, leaving 694 OTUs present in the 49 samples. After removal of Metazoa alignments, 587 OTUs were used for further analysis to determine the protist community. Identification up to genus level was accepted as species annotations were generally poor. Higher taxonomic levels included family, class, order, phylum and kingdom level. For taxa that could not be further identified, the previous higher taxonomic level was adopted and additions to the name were attached (e.g. unclassified) and counted as a different taxon on the respective taxonomic level.

### Statistical analysis

All statistical analyses were conducted in R, version 3.5.0 (R Core Team, 2018). The following packages were used for visualization: ggplot2 (Wickham, 2016), dendextend (Galili, 2015), ampvis2 (Andersen *et al*., 2018), RColorBrewer (Neuwirth, 2014), gplots (Warnes *et al*., 2019) and gridExtra (Auguie and Antonov, 2017). For significance tests, the significance level was set at *P* < 0.05.

For identification of significant abiotic correlations to our OTU abundance table, which was normalized to the total number of reads per sample, temperature, salinity, Secchi depth, tide and sunshine duration as well as silicate, nitrate, ammonium and phosphate concentrations were added to a “Constrained Ordination Model”. This model was based on an ANOVA-like permutation test for canonical correspondence analysis (CCA) to assess the significance of the constraining factors, by testing for single term additions (Oksanen *et al*., 2019). Single variables were chosen by their significance and added to the next step in the model, before the next significant variable was added in the next step. If several variables were given as significant, the variable with the lowest Akaike information criterion (AIC) value was chosen first to minimize the information loss (Akaike, 1974).

After calculation of the alpha diversity of the different taxonomic levels, the proportion of unclassified taxa—taxa that could not be determined and assigned by the PR2 database—were summarized and compared. Non-metrical multidimensional scaling (NMDS) plots were created in vegan with Bray–Curtis dissimilarities to compare the community composition of the samples on different taxonomic levels (Oksanen *et al*., 2019). Hereby, the data were converted to presence–absence data at genus and at phylum level. Beta diversity was calculated on genus level using the betadiver function vegan (Oksanen *et al*., 2019) and Whittaker index (Whittaker, 1960). To visualize the matrix, it was converted into a cluster with the hclust function. The phases that were chosen after comparing the NMDS plot with the beta diversity clusters were then tested for significance with an Analysis of Similarities (ANOSIM). A distance matrix of the phases defined by the beta diversity analysis was compared to the significant environmental parameters separately as they were defined by the CCA using a Mantel test from the ade4 package (Dray and Dufour, 2007; Bougeard and Dray, 2018). For the dissimilarity matrices of the determined phases and of environmental parameters, a Euclidean distance metric was used. To determine the most abundant genera, further analysis was based on the relative abundance of the Illumina reads per sample. To calculate the relative abundance, the dataset was normalized to the total number of reads per sample. Here, the most abundant genera had a relative sequence abundance of more than 5% in at least one sample during the whole period.

**Fig. 1:**
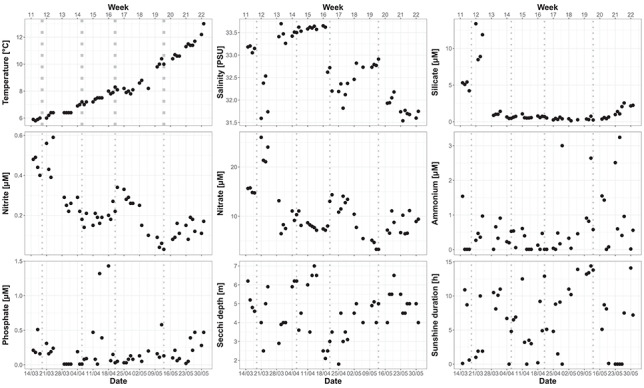
Profiles of temperature [°C], salinity, silicate [μM], nitrite [μM], nitrate [μM], ammonium [μM], phosphate [μM], Secchi depth [m] and sunshine duration [h] at Helgoland Roads LTER sampling station during spring 2016; vertical dotted lines indicate the different phases as defined by beta diversity analysis.

To define OTUs of interest, we conducted a Similarity Percentage analysis (SIMPER). The SIMPER analysis helps to identify those OTUs that contribute the most to the variation between the different phases. Using the phases that were defined based on the beta diversity calculation in the simper.pretty function (Steinberger, 2018), the OTUs with the biggest contributions to the similarity between two phases were identified. Hereby, OTUs that contributed less than 1% were removed. Afterwards the kruskal.pretty function (Steinberger, 2018) was used to find significant differences across the phases that were defined by the beta diversity calculation. The significant OTUs were then assigned to their respective genera and visualized as a heatmap. To find clusters of OTUs on presence–absence level, we used hierarchical cluster analysis with multiscale bootstrap in the parallel parPvclust function using the package pvclust (Suzuki and Shimodaira, 2006; Ryota Suzuki and Shimodaira, 2015). By development of a dendrogram with additional bootstrapping procedures, it is possible to calculate the significance of each cluster in the dendrogram. The number of bootstraps was elevated to 20 000 to minimize the standard error of the resulting clusters of OTUs. The calculation of distances for the hierarchical cluster was based on the asymmetric binary method, because the data are based on presence–absence level. For agglomeration, the complete linkage method (farthest neighbour clustering) was set. The pvpick function was used to find clusters with significant *P*-values. Support of data for these clusters was validated by manual estimation and comparison of the confidence interval to the respective *P*-values.

## RESULTS

### Environmental parameters and spring bloom succession as observed in the LTER

The water temperature at Helgoland Roads was 5.9°C on 15 March and gradually increased to 13°C until the end of May (Fig. 1, Supplementary Table SI). Salinity ranged from 31.5 to 33.7, showing fluctuations throughout the period. Silicate concentrations rose from 5.3 μM to reach a maximum on 21st March with 13.4 μM. At the end of March, concentrations declined and remained below 3 μM. Secchi depth varied throughout the sampling period between 1.8 and 7.0 m with several fluctuations. Daily sunshine duration varied greatly from day to day and ranged from 0 h of sunshine on 5th, 13th and 29th April and from 23rd May to 26th May up to 14.4 h of sunshine (12thMay).

**Fig. 2:**
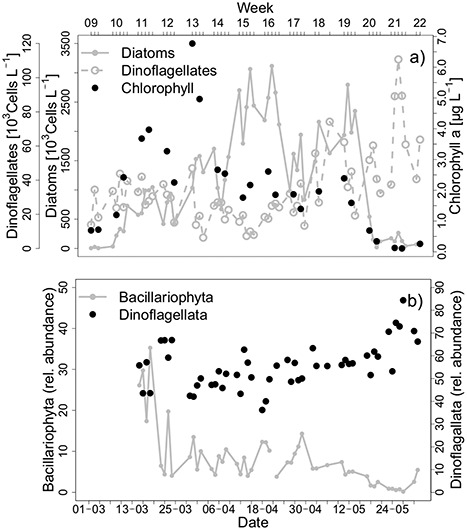
(a) Counts of diatoms and dinoflagellates [10^3^ cells L^−1^] and chlorophyll a [μg L^−1^] measured with HPLC at Helgoland Roads LTER station from March 1 to 31 May 2016; (b) relative abundance [%] of Bacillariophyta and Dinoflagellata from 15 March to 31 May 2016.

The LTER microscopic counts revealed a pattern, which resembled a typical spring phytoplankton succession with high diatom abundances, followed by a peak in dinoflagellates (Fig. 2a). Diatoms showed highest abundances (3116*10^3^ cells L^−1^) from week 14 to 16 (April) as well as during week 19 (May) (2795*10^3^ cells L^−1^). Dinoflagellate total counts revealed a maximum abundance at the end of May (week 21) (111*10^3^ cells L^−1^). In the beginning of March, HPLC chlorophyll a (Fig. 2a) was below 1.00 μg L^−1^ and increased to reach a first peak on 17th March (3.97 μg L^−1^). In contrast to the diatom maximum peak, chlorophyll a reached its peak on 29th March (week 13) with 6.77 μg L^−1^. Afterwards the concentration gradually declined with two maxima interrupting this trend on 19th April and 10th May, at 2.61 and 2.39 μg L^−1^, respectively.

### General description of the sequencing dataset

**Fig. 3:**
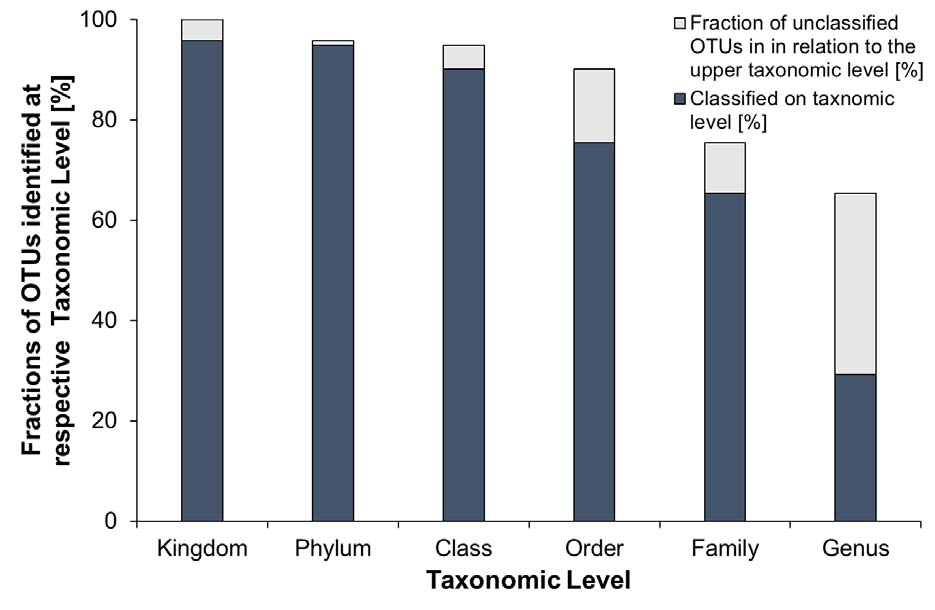
Fractions of OTUs identified at respective taxonomic level: dark grey indicates that identification on the respective taxonomic level was successful, light grey indicates that identification information did not go beyond the previous level; it includes all unclassified taxa (marked with a suffix_unclassified) and taxa where monophyly could not be insured (marked with a suffix_X according to the database).

After quality control, 587 OTUs were assigned to 21 phyla. Identification was conducted up to genus level (Fig. 3). Based on the total number of OTUs that was analysed, approximately 96% could be assigned at kingdom level. Assignment at phylum and class level was possible with more than 90% of the OTUs. At order and family level, 76 and 65% of all OTUs could be assigned, respectively. Most genera were represented by several OTUs. Examples are the dinoflagellate *Gyrodinium*, which was represented by nine OTUs, or the diatom *Chaetoceros*, which was assigned to seven OTUs. Overall, reliable identification at genus level was possible for only 29.3% of OTUs (83 genera), which indicates that the biggest information gap regarding taxonomic assignments occurs between family and genus level.

### Temporal dynamics in the community

**Fig. 4:**
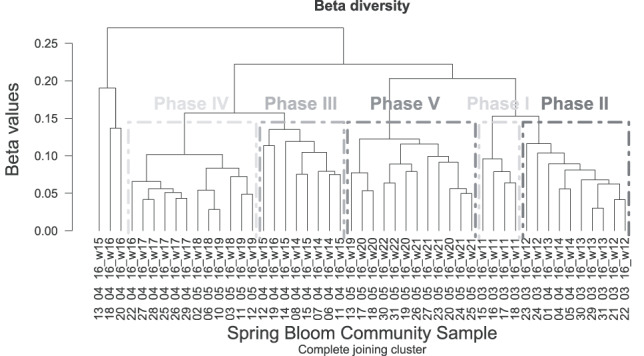
Beta diversity of the different samples during spring 2016. It was calculated using the betadiver function (vegan package) and Whittaker index; visualization of the matrix was done with the hclust function.

As shown in the 2D NMDS plots of community dissimilarities at presence–absence level (See online supplementary Fig. S1 for a colour version of this figure), a temporal pattern was found at genus level. However, the different communities are not visible at phylum level, since all phyla are represented by several genera that are always present. In general, beta diversity revealed a maximum species turnover of ~25% (Fig. 4). Five different phases could be identified during the spring bloom: phase 1 during week 11, phase 2 from week 12 to week 14, phase 3 from week 14 to week 16, phase 4 from week 16 to week 19 and phase 5 from week 19 to week 22 (see also Supplementary Table SI). The ANOSIM confirmed the significance of these clusters (*R* = 0.7, significance = 0.001).

**Fig. 5:**
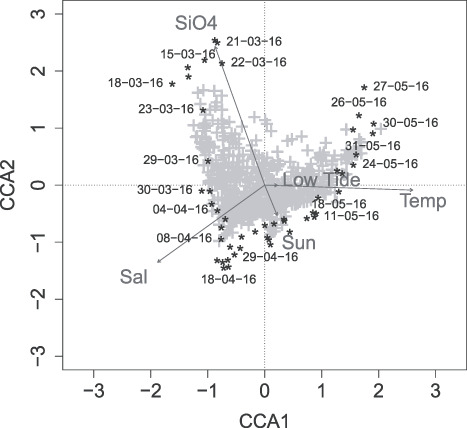
Canonical correspondence analysis (CCA) of the samples (black asterisks with sampling date) including abiotic factors in dark grey: temperature (Temp), salinity (Sal), silicate (SiO_4_), sunshine duration (Sun) and tide (Low Tide); OTUs in a light grey plus symbol, 37.9% of total inertia, could be explained by all variables, CCA1 explained 17.5% of the variance and CCA2 explained 10.5%.

For this spring bloom period, temperature (AIC = 251.73, *P* = 0.005) was found to be the most important environmental parameter based on the CCA model, followed by silicate (AIC = 247.26, *P* = 0.005), salinity (AIC = 245.95, *P* = 0.005), sunshine duration (AIC = 245.75, *P* = 0.005) and tide (AIC = 245.66, *P* = 0.005). Other parameters tested in the model that were not significant were nitrate, phosphate, ammonium and Secchi depth. The CCA plot (Fig. 5) indicated that at the beginning of the study period, the community was mostly correlated with silicate concentration. During April, this correlation shifted towards salinity which increased in April. Especially samples from the end of April and beginning of May were correlated to sunshine duration and low tide (information on tides can be found in Supplementary Table SI). The strongest correlation for the May community was with higher temperature.

**Fig. 6:**
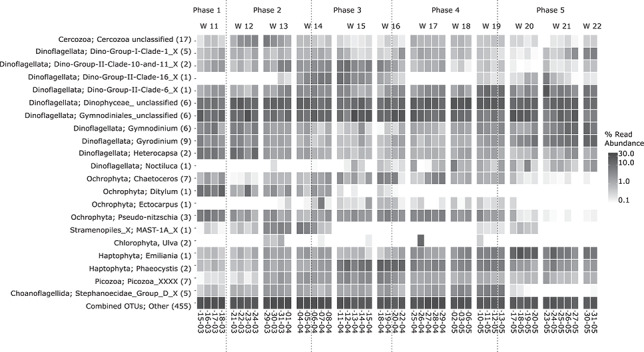
Most abundant genera from 15 March to 31 May 2016. Number of OTUs per genus indicated in parentheses. Shown are all genera with a relative sequence abundance of more than 5% in at least one sample during the whole timeframe.

The follow-up Mantel test revealed that the environmental factors temperature (*r* = 0.5738, *P* = 0.001) and salinity (*r* = 0.3483, *P* = 0.001) were significantly correlated to the beta diversity patterns, while silicate, sunshine duration and tide were not. Especially in phase 3, high variations in the community assemblage could be observed. Compared to the other phases, there were greater daily fluctuations in the community composition during phase 3. Three samples (13th, 18th, 20th April), which were taken during the same period, where the community of phase 3 was identified, showed higher variations in community composition and therefore could not be assigned to any phase.

SIMPER analysis showed that 53 OTUs explained at least 1% each of the variation between the five phases. With a Kruskal–Wallis test, 37 of these OTUs were found to be significantly different (Supplementary Table SII). These OTUs were from six different phyla and 28 genera, respectively. The number of contributing OTUs was increasing with later phases, and the highest number was found for phase 5 (15 OTUs). In total, 21 genera of 8 different phyla were found as most abundant (Fig. 6). Out of these 21 genera, 10 could not be assigned at genus level and 10 genera belonged to dinoflagellates. Unclassified Gymnodiniales and unclassified Dinophyceae OTUs contributed the most to the communities during all phases. The Ochrophyta genus *Ditylum,* followed by *Pseudo-nitzschia*, also contributed to the change in the overall community in all compared phases (Supplementary Table SII). *Ditylum* had the highest relative abundance during phase 1 and was declining at a fast rate during phase 2 and absent beyond phase 3 (Fig. 6). When comparing phases 2 and 3, unclassified Dinophyceae were identified as the biggest contributor to changes in the community, followed by the dinoflagellate *Heterocapsa* and heterotrophic Marine Stramenopiles-1A (MAST-1A). Regarding phase 3, 12 out of 16 OTUs, which contributed to the variation when compared to phase 4, belonged to dinoflagellate genera. At the end of phase 3 to the beginning of phase 4, a peak of *Phaeocystis* sp. abundances could be observed. Abundances of the nanoplanktonic coccolithophore *Emiliania* sp. rose to a peak during phase 4, with approximately 30% in relative abundance on 18 May 2016. This single *Emiliania* OTU had the biggest influence on the changes in community from phase 4 to phase 5. Over the complete sampling period, the same *Emiliania* OTU was found to have the largest influence when comparing phases 1 and 5, as abundance was increasing during the sampling period.

### Community structure and diversity of pico- and nanoplankton

Based on all 587 OTUs, the first phase consisted of the highest proportion of autotrophs and mixotrophs (on average ~60% in total) and 40% of heterotrophs (see Supplementary Table SIII for summarized suggested trophic modes; trophic modes were defined based on the taxonomy and known information from literature as Gómez (2012), Kubiszyn *et al*. (2014) and the Tara Oceans Database W4 from the Companion Website of the article of de Vargas *et al*. (2015); if the last identified taxon was on a higher taxonomic level, we assumed the likelier/more frequent trophic mode when suitable, otherwise no trophic mode was assigned.). For all other phases, heterotroph OTUs contributed the most with over 50%. Ochrophyta (See online supplementary Fig. S2 for a colour version of this figure), which were mostly represented by autotroph Bacillariophyta (diatoms, See online supplementary Fig. S3 for a colour version of this figure), were most abundant in phase 1. Single genera like *Chaetoceros* or *Pseudo-nitzschia* were also abundant during phases 3 and 4 (Fig. 6). During phase 5, diatom abundances were always low (<10%).

In accordance with the most heterotrophic phases 2–5, Dinoflagellata (See online supplementary Fig. S2 for a colour version of this figure) had consistently the highest relative abundances during the whole period, with relative abundances ranging from 36.2 up to 84.4%. Highest abundances were reached in phase 5. Three different classes of dinoflagellates (Dinophyceae, Noctilucophyceae and Syndiniales) could be identified (See online supplementary Fig. S3 for a colour version of this figure). Whereas Dinophyceae and Noctilucophyceae consist of mostly bigger sized dinoflagellates, Syndiniales consist of mostly picoplanktonic parasites. Dinophyceae were the biggest contributors to the community for most days, followed by Syndiniales, which were the biggest contributor in phases 3 and 5. The high contribution of dinoflagellate taxa was also visible in the number of taxa that were most abundant during this timeframe.

The next most important phylum was Haptophyta (See online supplementary Fig. S2 for a colour version of this figure). This pico- and nanoplanktonic phylum increased steadily in abundance during phases 1 and 2. High abundances with a maximum of 32.2% started from phase 3 onwards until the end of phase 5. Other phyla (See online supplementary Fig. S2 for a colour version of this figure) included the heterotroph Cercozoa (mostly unclassified), which showed high abundances during phase 2 (maximum 19.9%), but were generally low (<10%) before and after this period. Heterotrophic Stramenopiles represented by the pico- and nanoplanktonic MAST (See online supplementary Fig. S3 for a colour version of this figure) were mostly present during phases 2, 3 and 5 (24 different genera, in total 37 OTUs). During phase 2, a maximum abundance of 15.7% was reached, whereas during phase 5 the highest abundance was below 8%. Nine out of 21 phyla always had relative abundances below 1% (See online supplementary Fig. S2 for a colour version of this figure).

Based on presence–absence data, 33 significant clusters of OTUs were detected with hierarchical cluster analysis using 20 000 bootstraps (Supplementary Table SIV). The 33 clusters, representing a community of correlated OTUs, included 229 OTUs (39% of OTUs). Twenty-one of these clusters could be validated with the *P*-value being inside the confidence interval. Only four clusters had a relative abundance above 5% (Supplementary Table SV). The biggest cluster (cluster 4) included 79 OTUs. Except for four OTUs, the cluster consisted only of OTUs that were present in every sample and therefore during all phases. Fifty-two genera, belonging to 12 different phyla, could be found in this cluster. The biggest contributors were unclassified Dinophyceae and several pico- and nanoplanktonic MAST groups (14 OTUs). Another big and diverse cluster was cluster 31 with 11 OTUs, which were mainly found during phases 1 and 2. It included OTUs identified as Cercozoa, Ochrophyta (3 OTUs each), Stramenopiles_X (2 OTUs), Choanoflagellida, Ciliophora and Dinoflagellata (1 OTU each). The 12 significant clusters, where the confidence interval did not support the existence of the clusters, included between two and seven OTUs each. For example, six OTUs were part of cluster 22. Herein, two OTUs were unclassified Eukaryotes and four OTUs belonged to Hacrobia. Three nanoplanktonic cryptophytes (*Falcomonas* sp., *Teleaulax* sp., *Plagioselmis* sp.) and *Leucocryptos* sp. clustered together. Reads for these OTUs were available in phase 1, partially phase 4 and in phase 5.

## DISCUSSION

In this work, we could gather new information on several small-sized eukaryotic microbes. We identified nano- and picoplankton such as several Syndiniales (Dino-Groups) and MAST groups, *Phaeocystis* sp. and *Emiliania* sp., which contributed to the communities with high abundances. Additionally, we observed that our sequence assemblage was dominated by dinoflagellates, in contrast to the microscopic count data, and a peak of diatoms was not observed in the dataset.

### Environmental parameters and spring bloom succession as observed in the LTER

Our environmental conditions were mostly in accordance with the general pattern described by Wiltshire *et al*. (2015). While temperature and sunshine duration increased during our sampling period, salinity showed abrupt short-term changes. Higher salinity during our sampling indicates either a decreasing influence of riverine inputs or a bigger influence of Atlantic-driven waters during this time. As salinity was falling gradually, the increase in freshwater sources appears more likely. Wiltshire *et al*. (2015) stated that salinity reduction in spring happens mainly due to riverine input in late winter. As a result of incoming water masses, high concentrations of nutrients can be advected into the Helgoland Roads sampling site (Callies and Scharfe, 2015). In addition to biological nutrient cycling, change of water masses therefore can cause shifts in nutrient concentrations.

#### Comparison of spring bloom conditions regarding diatom and dinoflagellate occurrence

According to the LTER total counts, diatoms were much more abundant than dinoflagellates, and dinoflagellates reached their highest abundances after diatom abundances declined. This phenomenon is in accordance with previous literature from Helgoland Roads as well as other coastal European and North American regions (Lewandowska et al., 2015; Wiltshire et al., 2015; Carstensen et al., 2015). For the Western English Channel the spring diatom bloom is mostly followed by *Phaeocystis*, coccolithophorids and dinoflagellates (Widdicombe *et al*., 2010). However, for single regions the disappearance of a typical diatom spring bloom has been reported (Nixon *et al*., 2009).

The early peak in chlorophyll a measured by HPLC might be caused by picoplanktonic autotrophs (Knefelkamp, 2009) or the simultaneous high abundance of unclassified Cercozoa. Here, heterotrophic Cercozoa could have ingested chlorophyll-containing cells, or the Cercozoa were represented by chlorarachniophytes, which contain chloroplasts (Ishida *et al*., 1999). Also, it has to be noted that chlorophyll a sampling frequency was lower (two times a week), compared to the LTER counting data (five times a week).

If we compare the sequencing abundances regarding diatom and dinoflagellate abundances to the LTER total counts, we do not find a good match, even though the sampling frequency was similar and the high sampling frequency minimizes the chance that we missed individual abundance peaks that were seen in the microscopic counts. In addition, the typical decline in silicate concentration supports the presence of diatoms in high abundances. For example, as *Chaetoceros socialis* is known as a colonizing and mucous forming species; potential aggregation of cells needs to be taken into account (Riebesell, 1993). It is unclear to what extent aggregation potential of single species can influence the match in peak abundances for both methods, since aggregates in either sample might lead to overestimation.

With respect to diatoms in general (Ochrophyta), the highest abundances in our sequencing dataset were found in phase 1 at the beginning of the sampling period (week 11), with single genera also abundant during later phases. In total they did not show a distinct peak, but most genera found were in accordance with typical diatoms occurring in the area in spring (Hoppenrath, 2004; Wiltshire and Dürselen, 2004; Kraberg *et al*., 2015; Wollschläger *et al*., 2015). However, important species such as *Guinardia delicatula*, *Thalassionema nitzschioides* and *Odontella aurita*, which are known to have growth periods fitting to our sampling period, could not be found in high abundances. It has been shown that shifts in blooming periods and widening of occurrences of single species occurred in the past (Wiltshire *et al*., 2010; Schlüter *et al*., 2012), which could explain the absence of these species in our sequence assemblage. Comparison to the regular long-term microscopic counts revealed that *O. aurita* and *T. nitzschioides* were only reported for four and two times, respectively, during this timeframe. For *G. delicatula*, counts revealed that the species was mostly present from March to April (data not shown), which is in accordance with the sequencing results.

As the primer set used was engineered to better match contributions of diatoms and *Phaeocystis* sp. to the community, a sequencing bias should be unlikely. However, instead of a diatom-dominated community, our sequence assemblage was dominated by several dinoflagellate taxa. These included a wide diversity of large-sized species, but also potential parasites from different Syndiniales groups. The constant high abundance of dinoflagellates does not correlate with the LTER counts, where abundances steadily grew throughout the sampling period. Both datasets, however, showed the highest abundances in week 21.

So, what drives this conflicting information between microscopic counts and sequencing results? First, it has to be taken into account that the high abundances might be influenced by different dinoflagellate gene copy numbers. The generally high abundance of dinoflagellate genera was similar in previous studies. For instance, Massana *et al*. (2015) and Massana (2011) found mostly dinoflagellates including several parasitic Syndiniales in European coastal waters. Similar high abundances of Syndiniales and Gymnodiniales were found by Taylor and Cunliffe (2014) at the L4 coastal LTER station (Western English Channel). One issue is the use of relative abundances for comparison of communities that is influenced by gene copy numbers per cell, which differ greatly in between species. Several studies have emphasized the different rDNA copies among protist taxa like diatoms (Connolly *et al*., 2008) or dinoflagellates (LaJeunesse *et al*., 2005; Hong *et al*., 2016). Therefore, an approach based only on relative abundances is difficult to interpret. Several analyses in our study such as NMDS and OTU clustering were conducted at presence–absence level to avoid this phenomenon. However, one problem in using this approach was that most genera in this analysis were present at any time during the sampling period. In addition, since a comprehensive and reliable resolution at species level is not possible so far, it is necessary to include the relative abundances as well, if we want to see changes and relationships in the community. Furthermore, species that might be abundant at Helgoland and visible in the traditional long-term series when using microscopy might not be available in our dataset. Reasons for this could be the threshold we used, or a bias in DNA extraction, PCR and sequencing procedures. At the same time, it is possible that dinoflagellate occurrence in the environment is underestimated in microscopic studies, since several small-sized taxa cannot be identified.

Several factors might influence the reliability of sequence identification. Considerable difficulties and possible sources of biases include the use of target molecules (e.g. RNA, rDNA), regions (e.g. V4, V9) and databases like PR2 (Guillou *et al*., 2013) or SILVA (Pruesse *et al*., 2007). These databases are not of equal detail for different taxon groups. For example, identification on genus level for both databases was poor, and a direct comparison between PR2 and SILVA sometimes revealed contradictory results. In our dataset, barely any OTU could be differentiated down to species level, and a major proportion of OTUs could not be named at genus or higher levels either, indicating a considerable degree of hidden diversity in our dataset. For example, a high amount of big-sized dinoflagellate taxa could not be identified further, but might be identifiable using microscopy. However, for microscopy, too, it has to be noted that resolution at species level is mostly depending on taxonomic expertise, although resolution limits might not be as important for some easily identifiable taxa (Zingone *et al*., 2015). Moreover, the choice of target molecules and different regions influence the quality of the database alignment, since the genetic diversity of the target region might not be specific enough for identification at species level.

### Connections to environmental parameters and community dynamics

The CCA explained 37.9% of total inertia, which indicates that one or several additional factors, not yet taken into account, influenced the community at Helgoland Roads significantly. For example, Callies and Scharfe (2015) found hydrodynamic transport in regard to currents to be the most influential forcing parameter during spring, which was not considered in this study. The interplay between freshwater introducing influence by river discharge and marine water could only be discussed in regard to the rapid changes in salinity. In addition to the high influence of hydrodynamic transport and weather conditions, internal influences due to species interactions and grazing by zooplankton need to be taken into account as well in the future.

We observed five distinct phases in the spring bloom of 2016. As different analyses like the hierarchical clustering and NMDS showed, the community in phase 3 was having rapid changes compared to other phases. In addition, three samples that were taken in between samples from phase 3 could not be assigned to any phase, since they were more diverse. This indicates that additional communities might undergo rapid changes and would not be visible with a lower sampling frequency. It is noteworthy that this timeframe coincides with the maximum of the total counts of diatoms at the LTERsite.

The results regarding community composition showed that phases 1 and 5 were more similar to each other than the communities during phases 3 and 4. Comparing the beta diversity matrix with the environmental parameters, a significant correlation to temperature and salinity was shown. This result suggests that the contrasting environmental conditions like temperature differences did not inhibit the development of similar communities, which decreases the influence of temperature on community succession.

The most abundant genera were found in the OTUs with the greatest contributions by our SIMPER analyses. Since most abundant genera were available in our dataset during the whole sampling period, we can assume that these influence the community the most. Especially *Phaeocystis* sp. and *Emiliania* sp. could be identified as important blooming small-sized eukaryotic microbes. It has to be noted that our study is the first study using Illumina sequencing in this temporal resolution at Helgoland Roads. Therefore, it is not possible to compare our findings with sequencing data from previous years. However, several campaigns and efforts have been made to sample certain taxa or neighbouring areas. In the following paragraphs, we try to compare these findings by several different molecular methods with our results regarding the different taxa and small-sized eukaryotes.

### Diversity of nano- and picoplankton taxa

A considerable amount of new information about the spring bloom community on nano- and picoplankton composition was gained through this study, providing new insights into heterotrophic and possible parasitic components of the microbial loop communities.

Nano- and picoplankton taxa such as Syndiniales (Dino-Groups), *Emiliania* sp., *Phaeocystis* sp. and Choanoflagellida groups (*Stephanoecidae* Group D) were found in relatively high abundances and showed a distinctive blooming pattern during spring. Out of these taxa, only *Phaeocystis* sp. is counted at Helgoland Roads currently, while coccolithophorids like *Emiliania* and choanoflagellates cannot be identified on genus level. For other regions of the North Sea especially *Phaeocystis* and coccolithophorids are already known to be important compartments of the spring bloom community (Widdicombe *et al*., 2010). Despite their small cell size, *Phaeocystis* sp. are resistant against grazing by small-sized copepods due to their forming of gelatinous colonies and production of deterring chemicals, while different microzooplankton such as ciliates and heterotrophic dinoflagellates are known to feed on single *Phaeocystis* cells and on colonies (Hamm, 2000; Stelfox-Widdicombe *et al*., 2004; Schoemann *et al*., 2005). A shift from diatom blooms to *Phaeocystis*-dominated blooms therefore would influence the grazing success of the known copepods such as *Acartia* spp. and *Temora* spp. and change the whole food web dynamics at Helgoland.

If we look at other heterotroph small-sized eukaryotic microbes like the MAST groups, which are also not included in the LTER, we found a high amount of OTUs, of which several were present in all samples and clustered within the biggest cluster (cluster 4). This cluster included most OTUs that were present during the whole sampling period and represents a diverse background community. Accordingly, Logares *et al*. (2012) and Massana *et al*. (2014), who used data from several stations from European coasts, found the biggest contributions of different MAST groups in the pico- but also in the nanoplankton fraction.

Furthermore, cluster 22 stood out with mostly Hacrobia OTUs. In general, the OTUs in this cluster appear to play a role in early and late spring (phases 1 and 5), hinting that they might be suppressed by blooming plankton fractions, such as other Hacrobia like *Phaeocystis* and *Emiliania*. The cryptophytes from this cluster coincide well with findings from earlier studies (Metfies *et al*., 2010). Further analysis by Medlin *et al*. (2017) identified *Teleaulax*, *Plagioselmis* and *Geminigera* spp. as possible important cryptophytes during the spring bloom. In accordance with our results, these genera were abundant during the early and late phases of our spring bloom, but did not significantly contribute to the statistical similarities.

Diverse communities, such as represented by cluster 31, included taxa, which belonged to diatoms, heterotrophic flagellates or ciliate taxa. For example, choanoflagellates, as part of the heterotrophic nanoplankton, are a big contributor to carbon cycling in marine food webs, since they are grazing on bacteria and detritus but are themselves food for larger predators (King, 2005). The present fungi or fungi-like organisms act as decomposers of organic matter but can also be parasites of autotrophic primary producers and control their growth (Jobard *et al*., 2010). For Helgoland, it has been found that selective grazing by microzooplankton is important for phytoplankton spring bloom development and the occurrence of ciliates is dependent on specific preys (Löder *et al*., 2011). As these taxa cluster occurred during the early phases, where we observed the highest diatom abundances, a similar relationship can be suggested for our study.

## CONCLUSION

In order to achieve new insights to the Helgoland Roads eukaryotic microbial community during spring, we analysed the sequence assemblage and identified main abiotic correlations to the community dynamics. We obtained several unexpected results, which should be addressed in future observations. Most prominently, we observed a low occurrence of diatoms in our molecular dataset, despite the high sampling frequency, which we expect to be mainly caused by methodological constraints. Instead, our assemblage was mainly dominated by dinoflagellate OTUs. We could identify several taxa that occur at Helgoland during the whole period. At the same time, a rapid phytoplankton succession was observed, with some taxa only making occasional appearances. In accordance with our aim, we could identify many small-sized eukaryotic microbes, which showed a distinctive blooming pattern such as *Emiliania* and *Phaeocystis*. Pico- and nanoplankton are part of a core community, vary in bloom timing and form community clusters. Taking into account the abiotic factors used in our analysis, temperature and salinity were the abiotic parameters with the biggest correlations to the microbial communities present during our sampling period. However, it needs to be mentioned that contrasting conditions in these parameters did not prevent similar communities to evolve. Also, there are still unknown variables, which also influence the community structure that have not been taken into account. Since previous knowledge relies on microscopy, such as the known diatom spring bloom peak, which could not be identified in our dataset, there is a need to compare methods in more detail to overcome this issue and identify gaps and possibilities of synergy effects of the different datasets.

### DATA ARCHIVING

Sequence data for this study have been deposited in the European Nucleotide Archive (ENA) at EMBL-EBI under accession number PRJEB37135 (https://www.ebi.ac.uk/ena/data/view/PRJEB37135), using the data brokerage service of the German Federation for Biological Data (GFBio, Diepenbroek *et al*., 2014), in compliance with the Minimal Information about any (X) Sequence (MIxS) standard (Yilmaz *et al*., 2011).

## Supplementary Material

SupplementaryTable_SI_fbaa017

SupplementaryTable_SII_fbaa017

SupplementaryTable_SIII_fbaa017

SupplementaryTable_SIV_fbaa017

SupplementaryTable_SV_fbaa017

SupplementalFigures_fbaa017

Supplementary_Material_fbaa017
