# Erratum to: Rapid succession drives spring community dynamics of small protists at Helgoland Roads, North Sea

**DOI:** 10.1093/plankt/fbaa029

**Published:** 2020-06-13

**Authors:** Laura Käse, Alexandra C Kraberg, Katja Metfies, Stefan Neuhaus, Pim A A Sprong, Bernhard M Fuchs, Maarten Boersma, Karen H Wiltshire

**Affiliations:** 1 ALFRED-WEGENER-INSTITUT, HELMHOLTZ-ZENTRUM FüR POLAR- UND MEERESFORSCHUNG, BIOLOGISCHE ANSTALT HELGOLAND, 27498 HELGOLAND, Germany; 2 ALFRED-WEGENER-INSTITUT, HELMHOLTZ-ZENTRUM FüR POLAR- UND MEERESFORSCHUNG, 27570 BREMERHAVEN, Germany; 3 HELMHOLTZ-INSTITUT FüR FUNKTIONELLE MARINE BIODIVERSITäT, 26129 OLDENBURG, Germany; 4 DEPARTMENT OF MOLECULAR ECOLOGY, MAX PLANCK INSTITUTE FOR MARINE MICROBIOLOGY, 28359 BREMEN, Germany; 5 UNIVERSITY OF BREMEN, 28359 BREMEN, Germany; 6 ALFRED-WEGENER-INSTITUT, HELMHOLTZ-ZENTRUM FüR POLAR- UND MEERESFORSCHUNG, WADDEN SEA STATION, 25992 LIST AUF SYLT, Germany

Corresponding editor: John Dolan

In the first version of this article, Figures 4 and 5 were mistakenly switched in the text. This has now been corrected. The publisher regrets the error.

