# Supplementary material for: Rapid succession drives spring community dynamics of small protists at Helgoland Roads, North Sea
Source: J Plankton Res. 2020 May 14;42(3):305–19. doi: 10.1093/plankt/fbaa017 (PMC7252501; doi:10.1093/plankt/fbaa017)
Supplement: SupplementalFigures_fbaa017 [file supplementalfigures_fbaa017.doc]

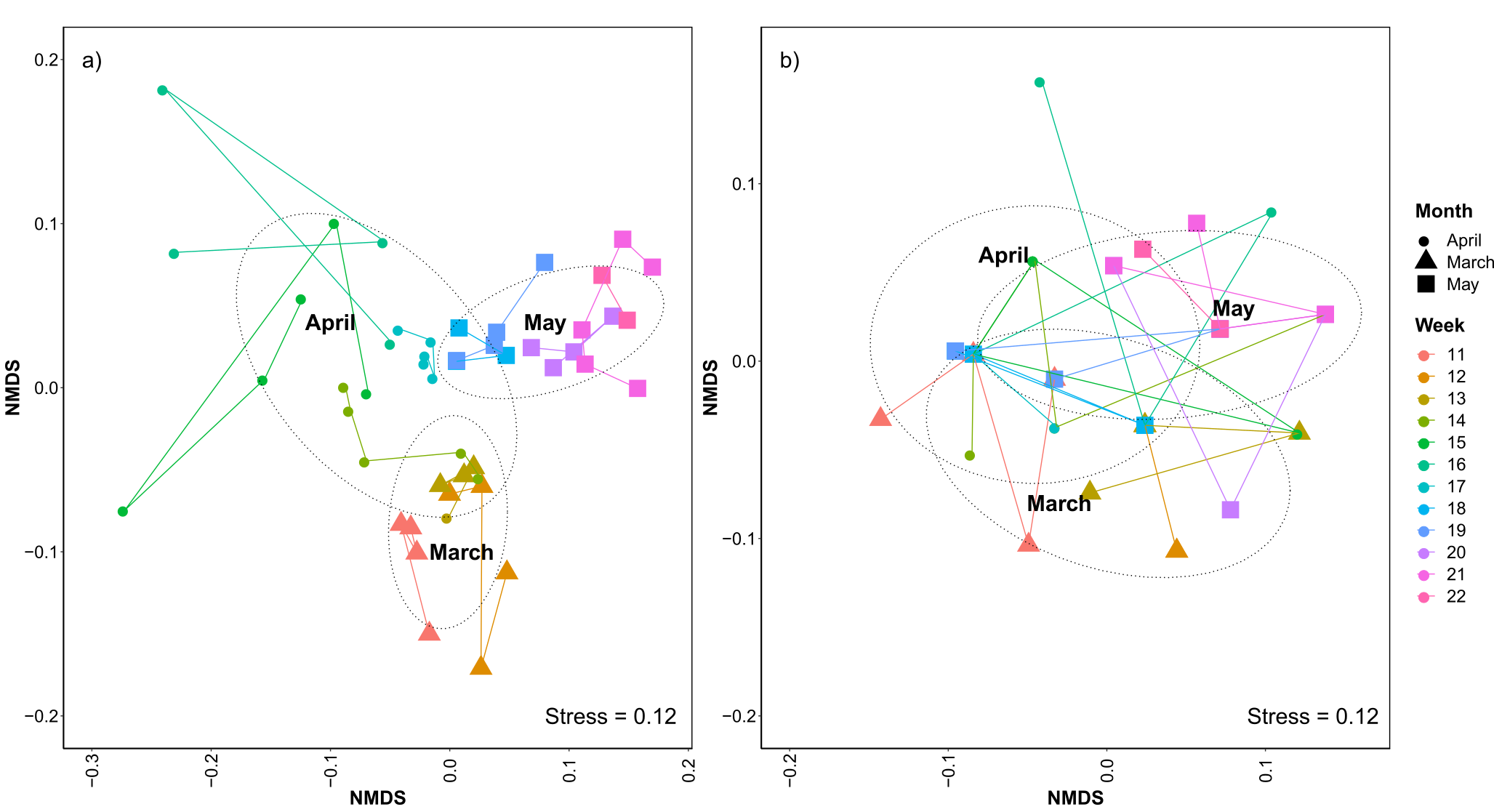


**Fig. SI.** Two-dimensional NMDS plots of community dissimilarities on a) genus level and b) phylum level based on presence-absence data of the respective taxon. Dissimilarity matrices are based on Bray-Curtis dissimilarities. Samples belonging to the same month are depicted by the same shape of points, colour coding shows weekly variations, lines connect the sampling days in weekly chronology. Ellipses were drawn at 80% confidence level.


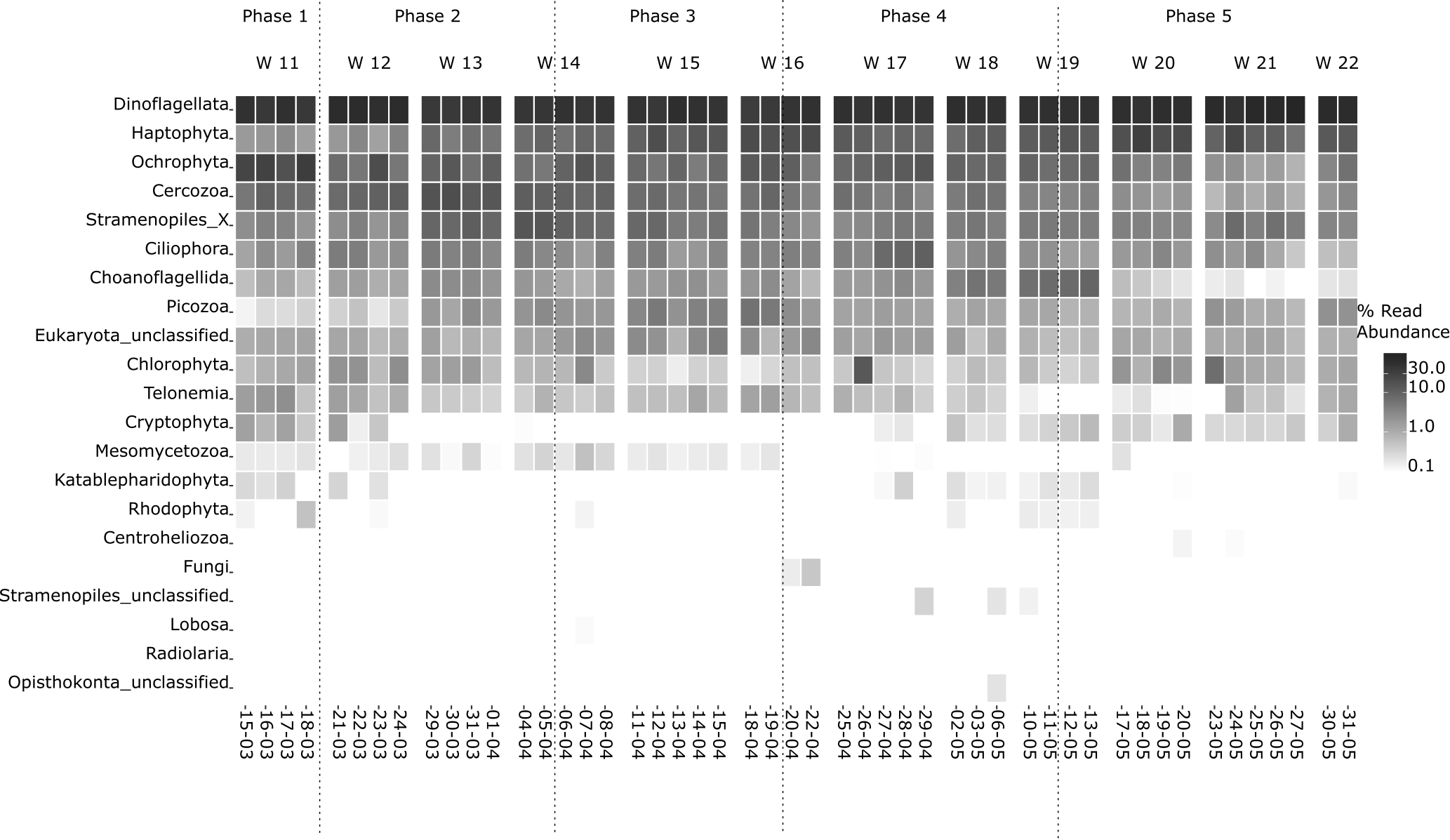


**Fig. SII.** Community composition on phylum level from March 15 to May 31, 2016, phyla are displayed according to the sum of their abundances in all samples.


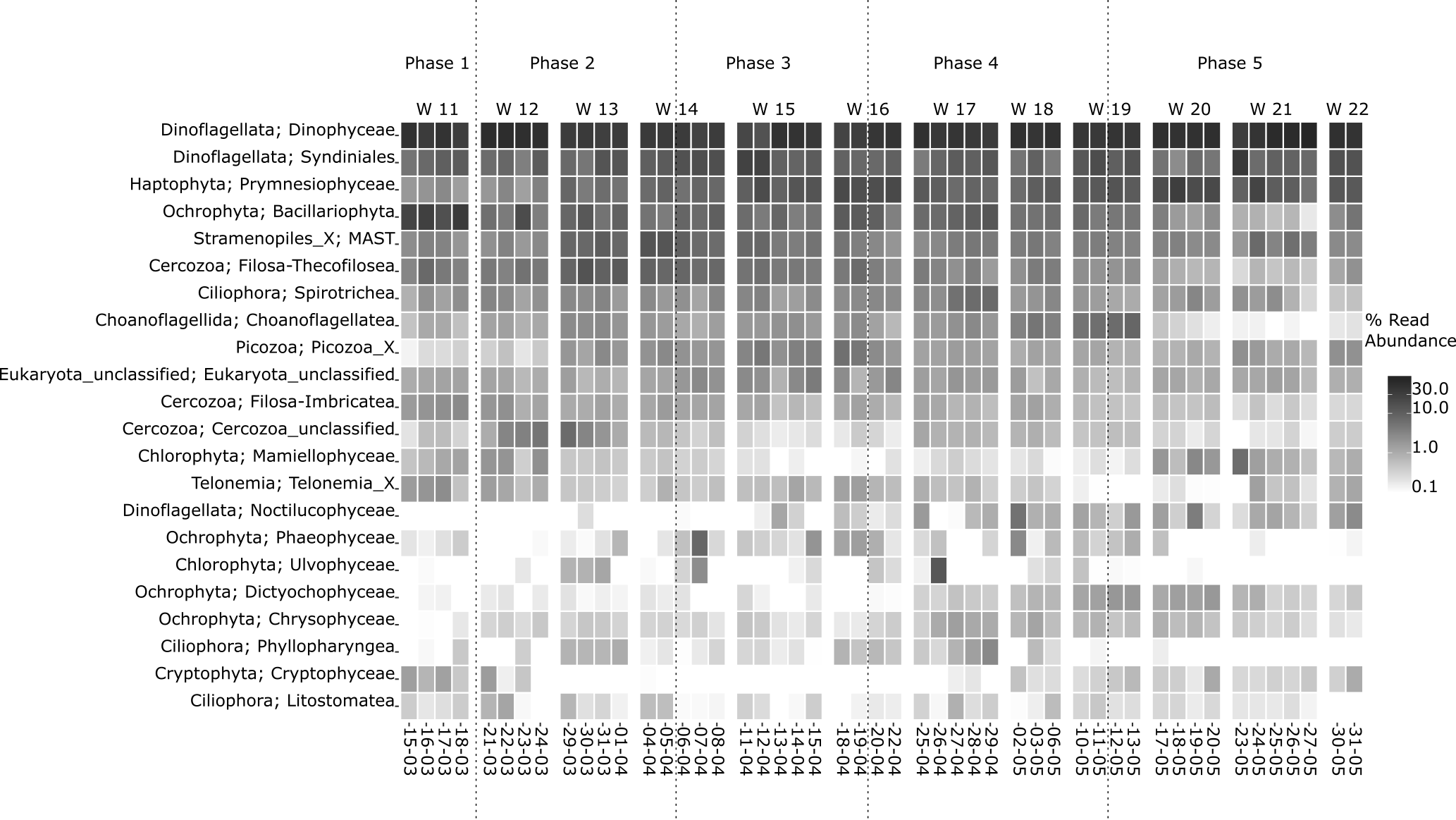


**Fig. SIII.** Community composition of the 22 most abundant classes from March 15 to May 31, 2016. First taxa is given on phylum level, second taxa on class level. Classes are displayed according to the sum of their abundances in all samples.
