# Supplementary material for: Rapid succession drives spring community dynamics of small protists at Helgoland Roads, North Sea
Source: J Plankton Res. 2020 May 14;42(3):305–19. doi: 10.1093/plankt/fbaa017 (PMC7252501; doi:10.1093/plankt/fbaa017)
Supplement: Supplementary_Material_fbaa017 [file supplementary_material_fbaa017.doc]

**Supplementary Material**

**Rapid Succession Drives Spring Community Dynamics of Small Protists at Helgoland Roads, North Sea**

# Materials and Methods

## Bioinformatics processing

Sequence processing, operational taxonomic unit (OTU) clustering, and annotation was done with an internally developed pipeline at the Alfred Wegener Institute as described below, wrapping common bioinformatics tools and "*GNU parallel*" (Tange, 2011) for fast and massive parallel workflow execution. Raw sequences were processed by the tool *Trimmomatic*, version 0.38 (Bolge*r et a*l., 2014), which scanned each sequence from 5' to 3' and trimmed the 3'-end if in a sliding window of 4 bp the average quality dropped below a Phred Q-score of 10. Sequences passing this filter were only retained if their paired-end partner also passed. *PEAR*, version 0.9.10 (Zhan*g et a*l., 2014) with default settings was used to merge the paired-end reads. Sequences which could not be merged were discarded.

To guarantee the same orientation, the sequences were filtered. Hereby, the sequence of the PCR forward primer had to occur before the sequence of the reverse complement of the reverse PCR primer. If sequences did not match this pattern, their reverse complement was also scanned. *Cutadapt,* version 1.17 (Martin, 2011) with the following settings was used for this task: for the forward primer sequence (19 bp long) a minimum sequence overlap of 16 bp and a maximum number of mismatches of 4 bp and for the reverse primer sequence (15 bp long) a minimum sequence overlap of 12 bp and a maximum number of mismatches of 3 bp were required to keep a sequence. Primer matching segments and additionally possible remaining artificial subsequences were finally truncated from the amplicon sequences. The remaining sequences were feature-filtered by *VSEARCH,* version 2.3.0 (Rogne*s et a*l., 2016): Sequences were discarded, i) if they were outside a 50 bp radius above or below the median length of the targeted amplicon (376 bp), i. e. below 326 bp or above 426 bp, ii) if they carried any ambiguity or iii) if the expected base error (sum of all base error probabilities) of a sequence was above 0.5.

Each sample was de-replicated individually (abundances of each amplicon kept in the sequence headers) and chimera were sample-wise predicted *de novo* by the tool *VSEARCH* (version 2.3.0) with default settings and removed from the sample files. Only samples with at least 10000 sequences after filtering were considered for further analyses (49 out of 50 samples). Cleaned sample files were pooled and now de-replicated in total while total amplicon abundances were kept in the sequence headers. Finally, about 4.3 million sequences (if one re-replicates the pooled amplicons) survived all filtering steps and were used as input of the OTU-clustering. OTU-clustering was done by the tool *Swarm*, version 2.1.8 (Mah*é et a*l., 2014, 2015) with default settings. Scripts needed to create the OTU-table from the *Swarm*-clustering output and from the abundance information in the headers of the chimera-cleaned sequence files were taken from *https://github.com/torognes/swarm/wiki/Working-with-several-samples*, adjusted and executed.

The most abundant amplicon of each OTU cluster was used as representative for the respective OTU. These sequences were annotated with the default classifier implemented in *mothur*, version 1.38.1 (Schlos*s et a*l., 2009) with the *Protist Ribosomal Reference database* (*PR2*), version 4.10 (Guillo*u et a*l., 2013) as reference set and a confidence cut-off of 90. The reference set was primer filtered and truncated the same way as the queries. The annotation of a representative sequence was used as annotation of the full OTU cluster and the annotation was added to the corresponding line of the OTU table.
